# MicroRNA-138 promotes acquired alkylator resistance in glioblastoma by targeting the Bcl-2-interacting mediator BIM

**DOI:** 10.18632/oncotarget.7346

**Published:** 2016-02-12

**Authors:** Nina Stojcheva, Gennadi Schechtmann, Steffen Sass, Patrick Roth, Ana-Maria Florea, Anja Stefanski, Kai Stühler, Marietta Wolter, Nikola S. Müller, Fabian J. Theis, Michael Weller, Guido Reifenberger, Caroline Happold

**Affiliations:** ^1^ Laboratory of Molecular Neuro-Oncology, Department of Neurology, University Hospital and University of Zurich, Zurich, Switzerland; ^2^ Neuroscience Center Zurich, University of Zurich, Zurich, Switzerland; ^3^ Department of Neuropathology, Heinrich Heine University Düsseldorf, Düsseldorf, Germany; ^4^ Institute of Computational Biology, Helmholtz Center Munich, German Research Center for Environmental Health, Neuherberg, Germany; ^5^ Molecular Proteomics Laboratory, Biological and Medical Research Center (BMFZ), Heinrich Heine University Düsseldorf, Düsseldorf, Germany; ^6^ Department of Mathematics, Technische Universität München, Garching, Germany; ^7^ German Cancer Consortium (DKTK), German Cancer Research Center (DKFZ) Heidelberg, partner site Essen/Düsseldorf, Germany

**Keywords:** chemoresistance, temozolomide, glioblastoma, miR-138, BIM

## Abstract

Glioblastoma is the most aggressive brain tumor in adults with a median survival below 12 months in population-based studies. The main reason for tumor recurrence and progression is constitutive or acquired resistance to the standard of care of surgical resection followed by radiotherapy with concomitant and adjuvant temozolomide (TMZ/RT→TMZ). Here, we investigated the role of microRNA (miRNA) alterations as mediators of alkylator resistance in glioblastoma cells. Using microarray-based miRNA expression profiling of parental and TMZ-resistant cultures of three human glioma cell lines, we identified a set of differentially expressed miRNA candidates. From these, we selected miR-138 for further functional analyses as this miRNA was not only upregulated in TMZ-resistant versus parental cells, but also showed increased expression *in vivo* in recurrent glioblastoma tissue samples after TMZ/RT→TMZ treatment. Transient transfection of miR-138 mimics in glioma cells with low basal miR-138 expression increased glioma cell proliferation. Moreover, miR-138 overexpression increased TMZ resistance in long-term glioblastoma cell lines and glioma initiating cell cultures. The apoptosis regulator BIM was identified as a direct target of miR-138, and its silencing mediated the induced TMZ resistance phenotype. Altered sensitivity to apoptosis played only a minor role in this resistance mechanism. Instead, we identified the induction of autophagy to be regulated downstream of the miR-138/BIM axis and to promote cell survival following TMZ exposure. Our data thus define miR-138 as a glioblastoma cell survival-promoting miRNA associated with resistance to TMZ therapy *in vitro* and with tumor progression *in vivo*.

## INTRODUCTION

Glioblastomas are the most aggressive primary brain tumors associated with a rapid and invariably fatal clinical course [1]. The current standard of care includes surgical resection followed by radiotherapy with concomitant and adjuvant chemotherapy with the alkylating agent temozolomide (TMZ/RT→TMZ) [2, 3]. Although TMZ prolongs progression-free and overall survival in glioblastoma patients, recurrence is inevitable because of infiltrative growth and inherent resistance to genotoxic stress. Even glioblastomas with favorable molecular profiles characterized by isocitrate dehydrogenase 1 or 2 mutation or O^6^-methylguanine DNA methyltransferase (*MGMT*) promoter methylation are prone to fail therapy.

MicroRNAs (miRNAs) are short, non-coding, single-stranded RNA molecules that function as negative post-transcriptional regulators of gene expression by binding to the 3′-untranslated region (3′UTR) of target mRNA, causing its cleavage or translational repression [4]. MiRNAs thereby regulate important cellular features such as growth, survival and death. Their expression is often deregulated in pathological conditions including cancer, where aberrant miRNA expression contributes to proliferation, invasion and resistance to apoptosis [5–7]. MiRNAs associated with TMZ resistance in glioblastoma include miR-195 [8], miR-9 [9], miR-125b [10], miR-136 [11], miR-181b [12], and miR-221/222 [13].

We have established a model of acquired resistance to TMZ in three well-characterized long-term glioblastoma cell lines (LTC) and investigated several candidate pathways of resistance to TMZ [14]. Here, we report a microarray-based screen for differential miRNA expression in pairs of parental and TMZ-resistant cultures. We identified a set of differentially expressed miRNA candidates and selected miR-138 for further analysis. miR-138 has been implicated in the pathogenesis of several cancers [15–17], including gliomas [18, 19], however, it has not been related to therapy resistance. Here, we demonstrate that miR-138 may mediate TMZ resistance by targeting Bcl-2-like protein 11 (BCL2L11), also known as BIM, a BH3-only apoptosis initiator from the Bcl-2 protein family [20].

## RESULTS

### miR-138 is upregulated in glioma cells with acquired TMZ resistance and in recurrent glioblastomas *in vivo*

**Figure 1:**
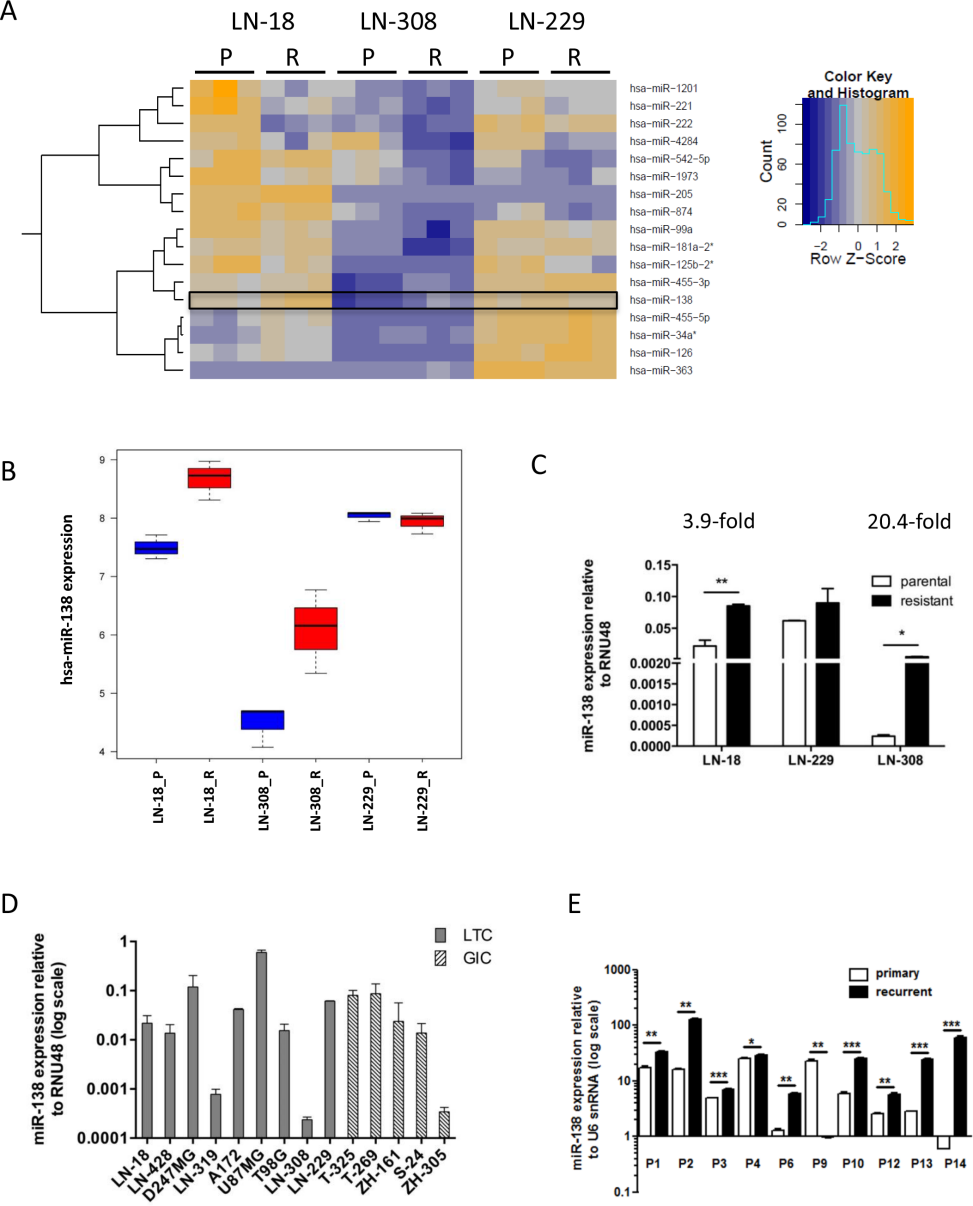
mir-138 is significantly upregulated in TMZ-resistant glioma cell lines and in recurrent glioblastoma (**A**) Heat-map of differentially expressed miRNA obtained by microarray-based expression profiling of parental (P) and TMZ-resistant (R) LN-18, LN-229 and LN-308 cells using GeneChip^®^ miRNA 2.0 arrays. Results were obtained from hybridization of three independent batches of each P and R cell line. Rows and columns were clustered hierarchically using the *hclust* method of R. The expression profile distances were calculated based on the Pearson correlation coefficients between miRNAs and samples. (**B**) Box plots showing upregulation of miR-138 expression in resistant versus parental cell lines of LN-18 and LN-308 but not LN-229 based on microarray data. (**C**) Real-time qPCR analysis for miR-138 expression in the paired P and R cells (*n* = 3, unpaired Student's *t*-test). (**D**) Relative baseline expression levels of miR-138 in LTC (gray columns) and GIC (striped columns) assessed by real-time qPCR (*n* = 3). (**E**) Real-time qPCR analysis of miR-138 expression in paired primary and recurrent glioblastoma tissues of 10 patients using U6 snRNA as reference (*p < 0.05; **p < 0.01; ***p < 0.001; paired Student's *t*-test).

Microarray-based miRNA expression profiling of parental and TMZ-resistant (TMZR) LN-18, LN-229 and LN-308 cells revealed several differentially expressed MiRNAs (Figure 1A), including several miRNAs previously implicated in TMZ resistance, such as mir-125b [10], miR-181 [12] or miR-221/222 [13]. We focused on MiRNAs upregulated in LN-308 cells since the mechanism of TMZ resistance in this cell line remained unclear [14]. We selected miR-138 for further investigations which showed increased expression in LN-18-resistant (LN-18_R) and LN-308-resistant (LN-308_R) cells (Figure 1B), and confirmed upregulation in these cells versus parental cells by real-time qPCR (Figure 1C). Next, we screened the baseline expression of miR-138 in a panel of 9 LTC and 5 GIC (glioma-initiating cells) lines (Figure 1D). There was no significant correlation of baseline miR-138 expression with sensitivity to TMZ in clonogenic survival assays either for all cell lines pooled (*r* = 0.25, *p* = 0.39) (Figure S1A), LTC alone (*r* = 0.03, *p* = 0.95) (Figure S1B), or GIC alone (*r* = 0.3. *p* = 0.68) (Figure S1C). We confirmed that the expression of miR-138 was increased in 9 of 10 paired tissues from primary and recurrent glioblastomas following TMZ/RT→TMZ, corroborating the potential significance of this miRNA in human glioblastoma *in vivo* (Figure 1E).

### miR-138 overexpression increases glioma cell proliferation

**Figure 2:**
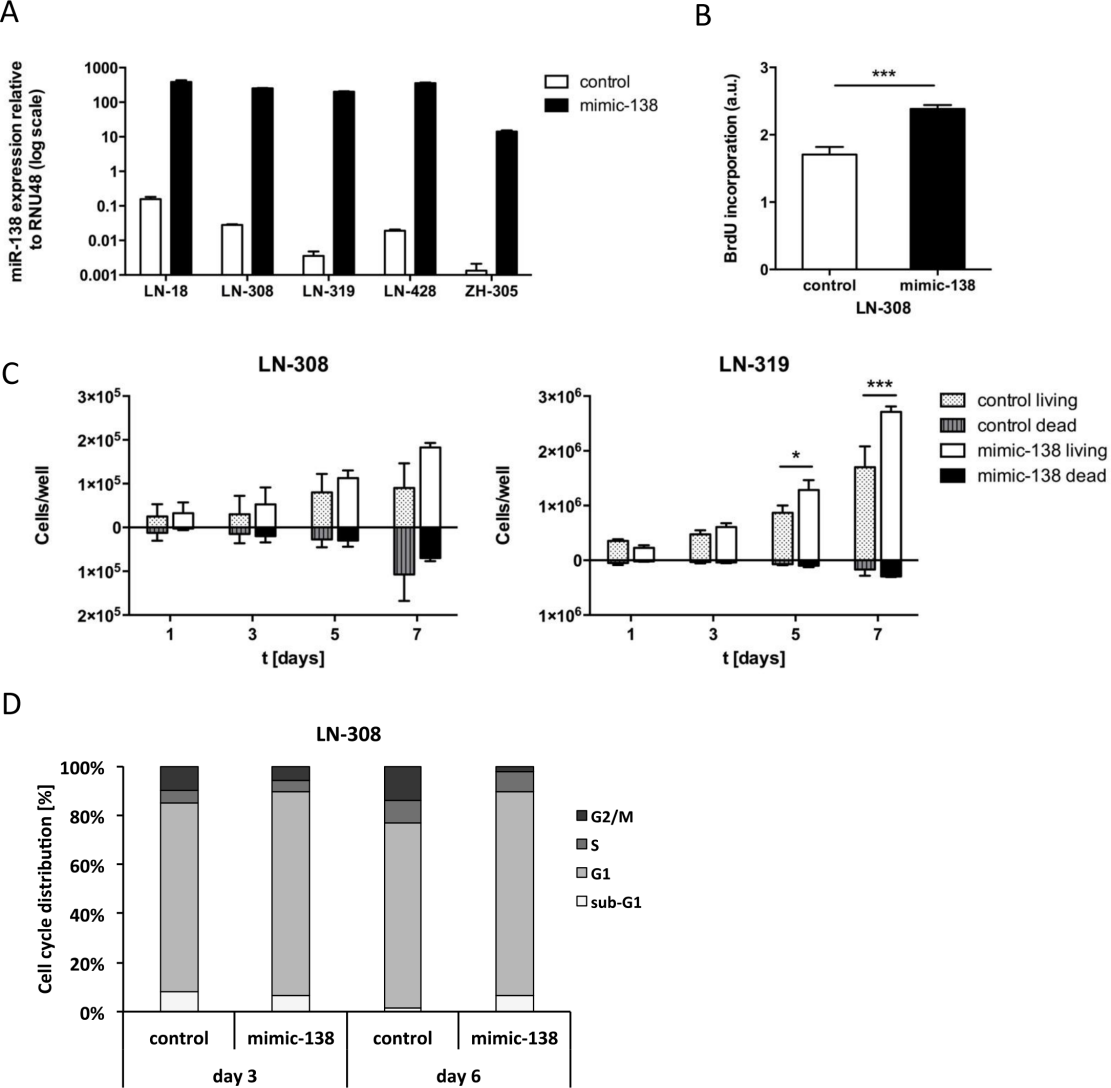
miR-138 induces proliferation of glioma cells *in vitro* (**A**) Transfection efficiency of mimic-138 in LN-18, LN-308, LN-319, LN-428 and ZH-305 cells was assessed by real-time qPCR of mimic and control transfected cells (*n* = 3). The final concentration of mimic/control was 50 nM in LTC and 100 nM in GIC. (**B**) BrdU incorporation assay was performed in LN-308 cells transfected with mimic-138 or control (50 nM) (***p < 0.001, unpaired Student's *t*-test). (**C**) LN-308 (left panel) or LN-319 (right panel) cells were transfected with mimic-138/control (50 nM) and proliferation and cell death were measured by counting trypan blue stained cells at days 1, 3, 5 and 7 after reseeding the transfected cells. Assay was analyzed by unpaired Student's *t*-test. (**D**) Flow cytometric cell cycle analysis of mimic-138/control (50 nM) transfected LN-308 cells was performed by PI staining at day 3 and 6 post-transfection.

Next we assessed the effects of miR-138 overexpression in LN-308 and LN-319 cells selected for their low baseline expression of miR-138 (Figure 1D, Figure 2A). miR-138 induced proliferation of LN-308 glioma cells as indicated by BrdU incorporation assay (Figure 2B), confirmed by trypan blue dye exclusion assays over time in both cell lines (Figure 2C). Cell cycle analysis by flow cytometry showed a reduced G2/M fraction in the miR-138 overexpressing cells at day 6 post transfection (Figure 2D).

### Overexpression of miR-138 induces resistance to TMZ and CCNU in glioma cells

**Figure 3:**
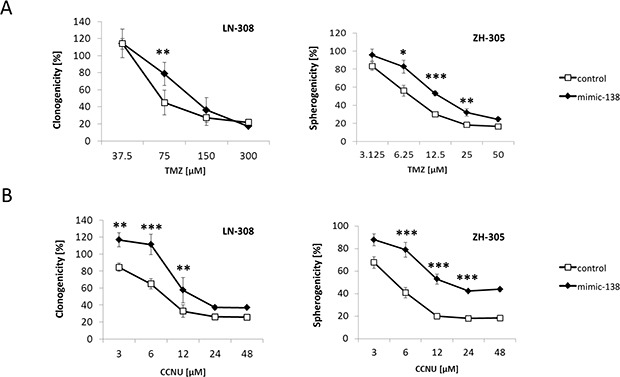
Overexpression of miR-138 confers TMZ and CCNU resistance in glioma cells (**A**) LN-308 and ZH-305 cells were transfected with miR-138 mimics or control and TMZ or (**B**) CCNU sensitivity was assessed by clonogenic survival assays in LTC and spherogenicity assays in GIC (concentration of mimic/control: 50 nM for LTC, 100 nM for GIC) (*p < 0.05, **p < 0.01, ***p < 0.001, unpaired Student's *t*-test).

In LN-308 and ZH-305 cells, chosen for their low baseline expression of miR-138, overexpression of miR-138 conferred protection from TMZ. The EC_50_ shifted from 40 to 120 μM in LN-308 and from 8 to 16 μM in ZH-305 (Figure 3A). In contrast, no such effect was seen in LN-18 or LN-428 cells (Figure S2A), which have higher baseline expression of miR-138 (Figure 1D). When miR-138-overexpressing LN-308 and ZH-305 cells were exposed to increasing concentrations of another alkylating agent, CCNU, a gain of resistance was also achieved (Figure 3B). No cross-resistance was observed to irradiation, to the topoisomerase II inhibitor etoposide, or to the vinca alkaloid vincristine (Figure S2B). To address whether the TMZ resistant phenotype is reversed by downregulation of endogeneous miR-138, we transiently transfected miR-138-expressing LN-18 and LN-229 cells with a specific miR-138 inhibitor or a non-targeting negative control molecule. Transfection efficacy was assessed by real-time qPCR (Figure S3A) and by fluorescent light microscopy (Figure S3B). Immunoblot analysis for expression of the miR-138 target SOX4 [17] in LN-229 cells confirmed the biological activity of miR-138 inhibition (Figure S3C). Conversely, the overexpression of miR-138 decreased SOX4 levels by 40% in LN-308 cells selected for their low baseline miR-138 expression. However, the inhibitor did not increase sensitivity to TMZ in LN-18, LN-18_R, LN-229 or LN-229_R cells (Figure S3D). We assume that upon miR-138 inhibitor application, residual endogenous levels of miR-138 are still high enough to preserve the primary resistant phenotype.

### Predicted miR-138 targets in mediating TMZ sensitivity – no role for activated leukocyte cell adhesion molecule (ALCAM)

ALCAM, an *in silico* predicted target of miR-138 [21], emerged as a candidate decreased in LN-18_R and LN-229_R cells from the microarray-based mRNA expression profiling (data not shown). According to The Cancer Genome Atlas (TCGA) online dataset [22], low expression levels of ALCAM correlate with shorter overall survival of glioblastoma patients (*n* = 504; average cut-off = 590.3; *p* = 5.2e−03) (Figure S4A). We confirmed a downregulation at protein level by immunoblot in LN-18_R and LN-229_R cells (Figure S4B). However, siRNA-mediated gene silencing of ALCAM (Figure S4C) did not confer TMZ resistance (Figure S4D), which indicates that ALCAM downregulation alone is not sufficient to mediate TMZ resistance in glioma cells.

### BIM is a direct target of miR-138 and modulates TMZ resistance

**Figure 4:**
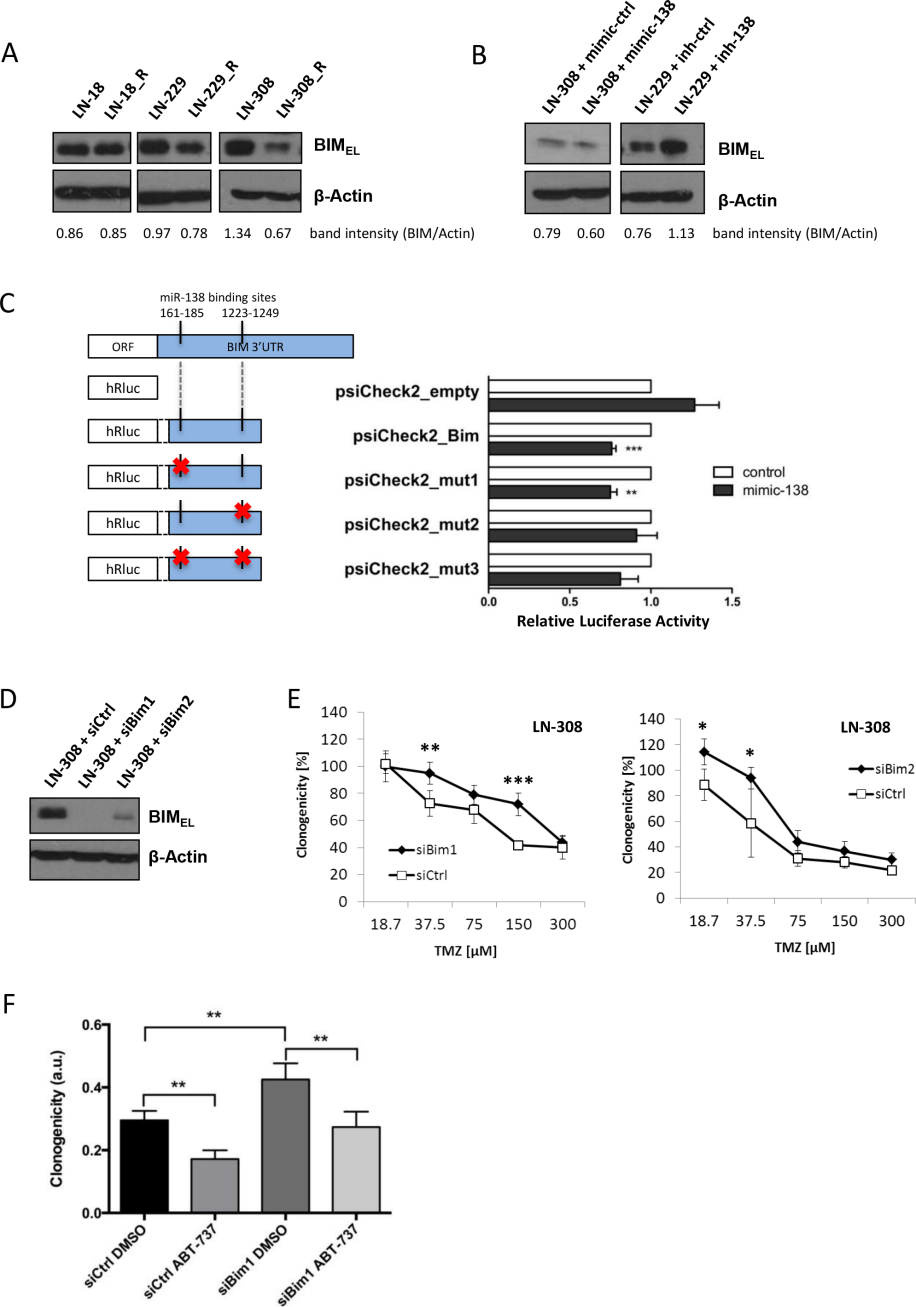
BIM downregulation via miR-138 confers TMZ resistance to glioma cells (**A**) Immunoblot for BIM detection in LN-18, LN-229 and LN-308 parental versus resistant (R) cells (**B**) and in LN-308 or LN-229 cells transfected with miR-138 mimic, inhibitor or the respective control (100 nM); lysates were collected 72 h post-transfection. Quantification of protein bands was performed by densitometry, and the values stated under each band represent the normalized value to the corresponding loading control signal. (**C**) Luciferase reporter assay in LN-308 cells co-transfected with psiCheck2 empty vector, psiCheck2_Bim vector or one of the three mutant control vectors (psiCheck2_mut1, psiCheck2_mut2 and psiCheck2_mut3) and miR-138 mimic or control (transfection performed with 100 ng vector and 100 nM mimic/control concentration). Left panel: schematic of the two miR-138 binding sites in the 3′UTR region of the BIM mRNA, and the content of each vector construct (red cross represents mutation in the recognition site). Right panel: representative luciferase reporter assay. (**D**) siRNA-mediated silencing of BIM in LN-308 cells confirmed by immunoblot analysis (siRNA concentration: 100 nM). (**E**) Clonogenic survival assay after TMZ exposure of LN-308 cells transfected with two different siBim constructs (siBim1 and siBim2) or with siControl (siCtrl) (100 nM). (**F**) Clonogenic cell survival assay after TMZ treatment (37.5 μM) in combination with ABT-737 (3.3 μM) or DMSO of siBim1 or siControl transfected LN-308 cells. All values were normalized to siCtrl cells treated with DMSO (solvent control) and TMZ-untreated cells.

We further investigated additional predicted miR-138 targets focusing on those that were found to be downregulated at the mRNA level by Affymetrix gene chip analyses of the three parental and resistant glioma cell lines. The predicted target BCL2L11 (or BIM) was found to be downregulated in all three resistant cell lines in the Affymetrix array. Accordingly, in the TCGA online dataset, low expression levels of BCL2L11/BIM correlate with shorter overall survival of glioblastoma patients (Figure S5). Down-regulation of BIM protein was confirmed by immunoblot analysis in LN-229_R and LN-308_R cells (Figure 4A). BIM has three predominant isoforms generated by alternative splicing, BIM_EL_ (extra-large), BIM_L_ (large) and BIM_s_ (small). These isoforms are ubiquitously expressed with a tissue-specific variation, where BIM_EL_ is the most abundant isoform. Only BIM_EL_ was detected in our glioma cells. This case of preferential expression of one BIM isoform in different cell types/tissues has been previously described [23, 24]. BIM levels were reduced or increased, respectively, in glioma cells transfected with miR-138 mimic or miR-138 inhibitor (Figure 4B). Luciferase reporter assays assessing miR-138 binding to the *BIM*-3′UTR region confirmed BIM as a direct target of miR-138. Additionally, control vectors carrying either each at a time or both mutated miR-138-binding sites in the *BIM*-3′UTR region revealed that the binding site 1223-1249 nt is more potently regulated by miR-138 (Figure 4C; Figure S6A). SiRNA-mediated *BIM* gene silencing (Figure 4D, Figure S6B) increased TMZ resistance in LN-308 cells (Figure 4E). To confirm the role of the BIM-related apoptotic pathway, we employed sub-lethal concentrations of ABT-737 (Figure S6C), a small-molecule BH3 mimetic that directly inhibits anti-apoptotic proteins Bcl-2, Bcl-xL and Bcl-w [25]. The resistant phenotype induced by *BIM* gene silencing was indeed reversed by ABT-737 (Figure 4F).

### TMZ treatment does not induce acute apoptosis, but modulates expression of Bcl-2 family proteins

**Figure 5:**
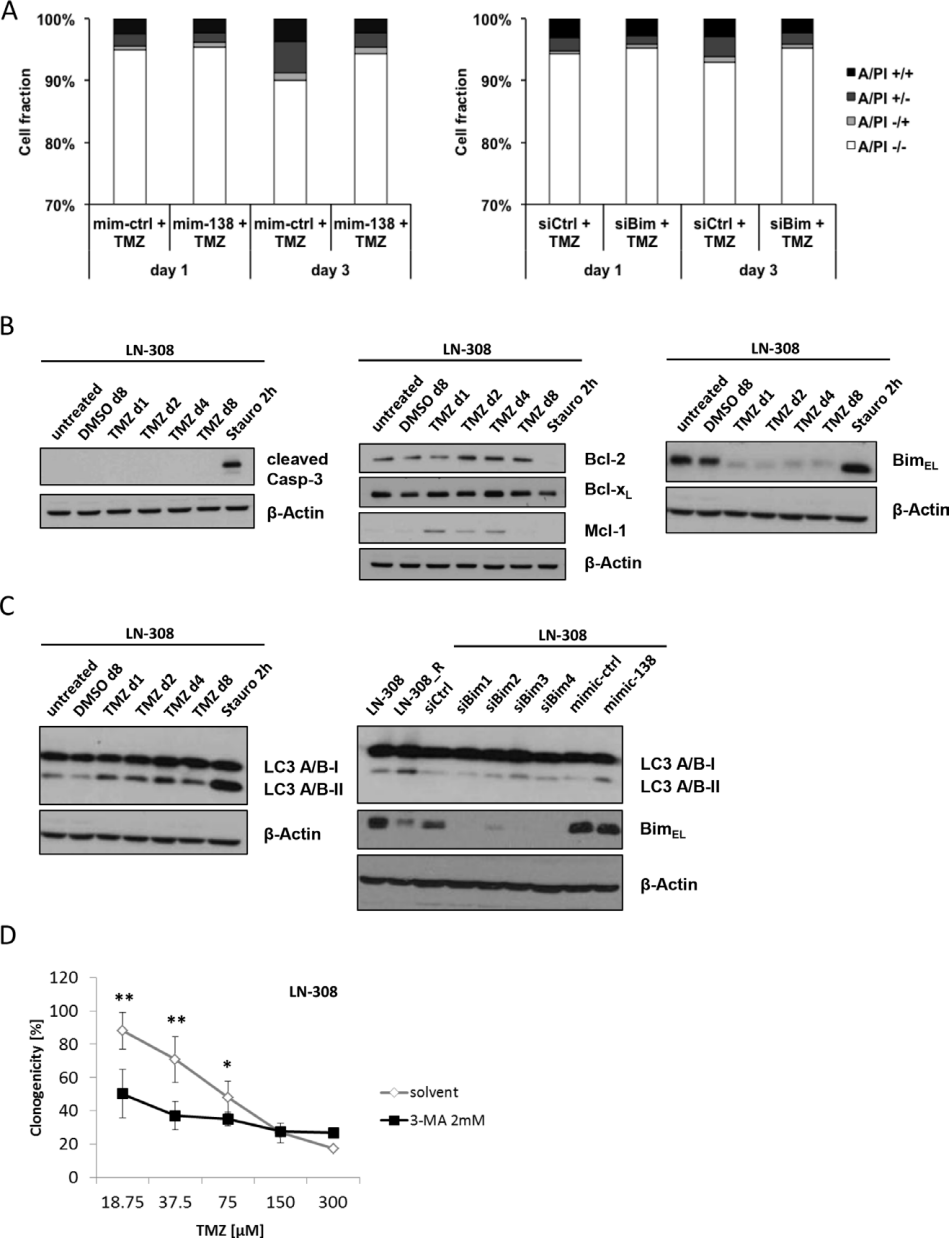
TMZ and miR-138 induce little apoptosis but cytoprotective autophagy (**A**) Annexin V/PI flow cytometric analyses of LN-308 mim-138/control (left panel) or siBim/control (right panel) transfected cells treated with TMZ (100 μM) 1 and 3 days later. (**B**) Immunoblot analysis of cleaved caspase 3 (Casp-3) levels in LN-308 cells at different indicated time points after DMSO (vehicle control), TMZ (100 μM) or staurosporin (Stauro, 2 μM) stimulation (left panel), and of Bcl-2, Bcl-x_L_, Mcl-1 and BIM levels in LN-308 cells at different indicated time points after DMSO (vehicle control), TMZ (100 μM) or staurosporin (Stauro, 2 μM) stimulation (middle panel, right panel). (**C**) Immunoblot analysis of LC3 A/B I and II levels in LN-308 cells at different indicated time points after DMSO (vehicle control), TMZ (100 μM) or staurosporin (Stauro, 2 μM) stimulation (left panel); immunoblot analysis of LC3 A/B I and II levels and BIM levels in LN-308 and LN-308_R cells; LN-308 cells were transfected with siCtrl or 4 different siBim molecules (100 nM), and mimic-ctrl (mim-ctrl) or mimic-138 (mim-138) molecules (50 nM), and cell lysates were collected 72 h after transfection (right panel). Quantification of protein bands was performed by densitometry for BIM levels in mim-ctrl and mim-138 transfected cells; values representing the normalized value to the corresponding loading control signal were 1.38 for mim-ctrl and 1.14 for mim-138, respectively (**D**) Clonogenic survival assay after TMZ exposure of LN-308 cells treated with the autophagy inhibitor 3-MA (2 mM) or solvent control.

BIM belongs to the BH-3 only group of pro-apoptotic Bcl-2 family members and is a direct regulator of the intrinsic cell death pathway. To assess whether TMZ protection of glioma cells by miR-138 over-expression or by *BIM* silencing involves cell death regulation, we performed flow cytometry cell death analysis by Annexin V/PI staining at days 1 and 3 after TMZ treatment. The overall amount of cell death induced by TMZ was low (up to 10% on day 3). Still, miR-138 mimic or siBim -transfected cells showed reduced cell death upon TMZ stimulation at day 3 (Figure 5A), suggesting that regulation of apoptosis via miR-138 and BIM is only partially involved in acquired TMZ resistance. In order to assess induction of apoptosis by a complementary approach, we checked for caspase 3 cleavage on protein level in LN-308 cells treated with TMZ, using staurosporin as a positive control. We failed to detect caspase 3 cleavage after a short period of TMZ treatment (1–2 days), as well as after an extended period of 4–8 days (Figure 5B). Next we investigated the protein levels of the pro-apoptotic protein BIM and of the anti-apoptotic Bcl-2 family members Bcl-2, Bcl-x_L_ and Mcl-1 after TMZ treatment. Interestingly, protein levels of Bcl-2 and Mcl-1 increased as early as 1 day after TMZ treatment, and in the case of Bcl-2, the protein levels were further elevated at later time points (day 2–8). BIM levels, however, decreased dramatically already after one day of TMZ treatment and remained constantly down up to 8 days after stimulation (Figure 5B), indicating an induction of a pro-survival signaling pathway mediated by the anti-apoptotic protein stabilization as well as BIM degradation rather than apoptotic cell death.

### Autophagy is induced by TMZ and miR-138, negatively correlates with BIM protein levels, and acts as a pro-survival pathway

Different pathways have been reported to promote survival or bypass cell death under stress conditions, including autophagy [26–29]. Accordingly, LN-308 were exposed to TMZ and activation of autophagy was measured by conversion of the light chain 3-I (LC3-I) to LC3-II, a marker for autophagy [30]. Indeed, TMZ treatment led to a strong increase in LC3-II levels over time (Figure 5C). Since BIM protein levels are negatively regulated by miR-138, and miR-138 expression in turn is associated with TMZ-induced resistance, we assessed the correlation between miR-138-dependent BIM regulation and autophagy in response to TMZ in glioma cells. Indeed, we recapitulated induction of autophagy in LN-308_R cells, where BIM levels were reduced (Figure 5C). More importantly, knockdown of *BIM* or transfection of miR-138 mimic led to enhanced autophagy as assessed by increased LC3-II production (Figure 5C). Band intensity quantification confirmed a reduction of BIM protein levels in the miR-138 mimic-transfected cells. To assess whether the induced autophagy in LN-308 cells plays a chemoprotective role, we performed clonogenic survival assays with TMZ in the presence or absence of the autophagy inhibitor 3-methyl adenine (3-MA). When autophagy was blocked, cells were sensitized to TMZ (Figure 5D), suggesting that autophagy is a pro-survival signal mediating TMZ resistance in glioma cells.

## DISCUSSION

Increasing evidence suggests that miRNAs play important roles in proliferation, invasion and migration as well as spontaneous and induced cell death in multiple cancer entities, including glioblastoma [31, 32]. Moreover, numerous studies have implicated altered miRNA expression in constitutive or acquired resistance to specific cancer treatments, such as irradiation or chemotherapy [33, 34]. Here we used microarray-based miRNA expression profiling of paired parental and TMZ-resistant glioma cell lines [14] to identify novel miRNA candidates potentially mediating resistance of glioma cells to TMZ, which together with radiotherapy constitutes the current standard of care in glioblastoma patients [3]. We provide evidence that miR-138 mediates acquired TMZ resistance in glioblastoma by regulating the apoptosis inducer BIM and autophagy.

miR-138 has been implicated in the promotion of growth and survival of glioma stem cells [18], however, it appears to have an opposite role as a tumor suppressor in other cancers [16, 35]. Upregulation of miR-138 in recurrent glioblastomas of patients progressing after radiotherapy and TMZ confirmed the potential *in vivo* relevance of our *in vitro* profiling approach (Figure 1E). We found no clear-cut association between baseline miR-138 expression levels and TMZ sensitivity of various glioma cell lines (Figure S1). This finding may partially be due to the overwhelming role of *MGMT* promoter methylation and expression in TMZ sensitivity [36], whose strong impact on alkylator resistance might override the effects of miR-138-mediated TMZ resistance in these cell lines. Moreover, miR-138 may be of particular importance in acquired rather than constitutive TMZ resistance, consistent with the observation that DNA damage induces expression of different microRNAs via transcriptional or post-transcriptional regulation [37].

We confirmed as previously described [18] that overexpression of miR-138 promotes cell cycle progression and drives glioma cell proliferation (Figure 2B–2D), but this is at odds with the report that miR-138 overexpression in glioma reduced cell proliferation *in vitro* and tumorigenicity *in vivo* through inducing a G1/S cell cycle arrest by inhibition of a EZH2-CDK4/6-pRb-E2F1 signaling loop [19].

**Figure 6:**
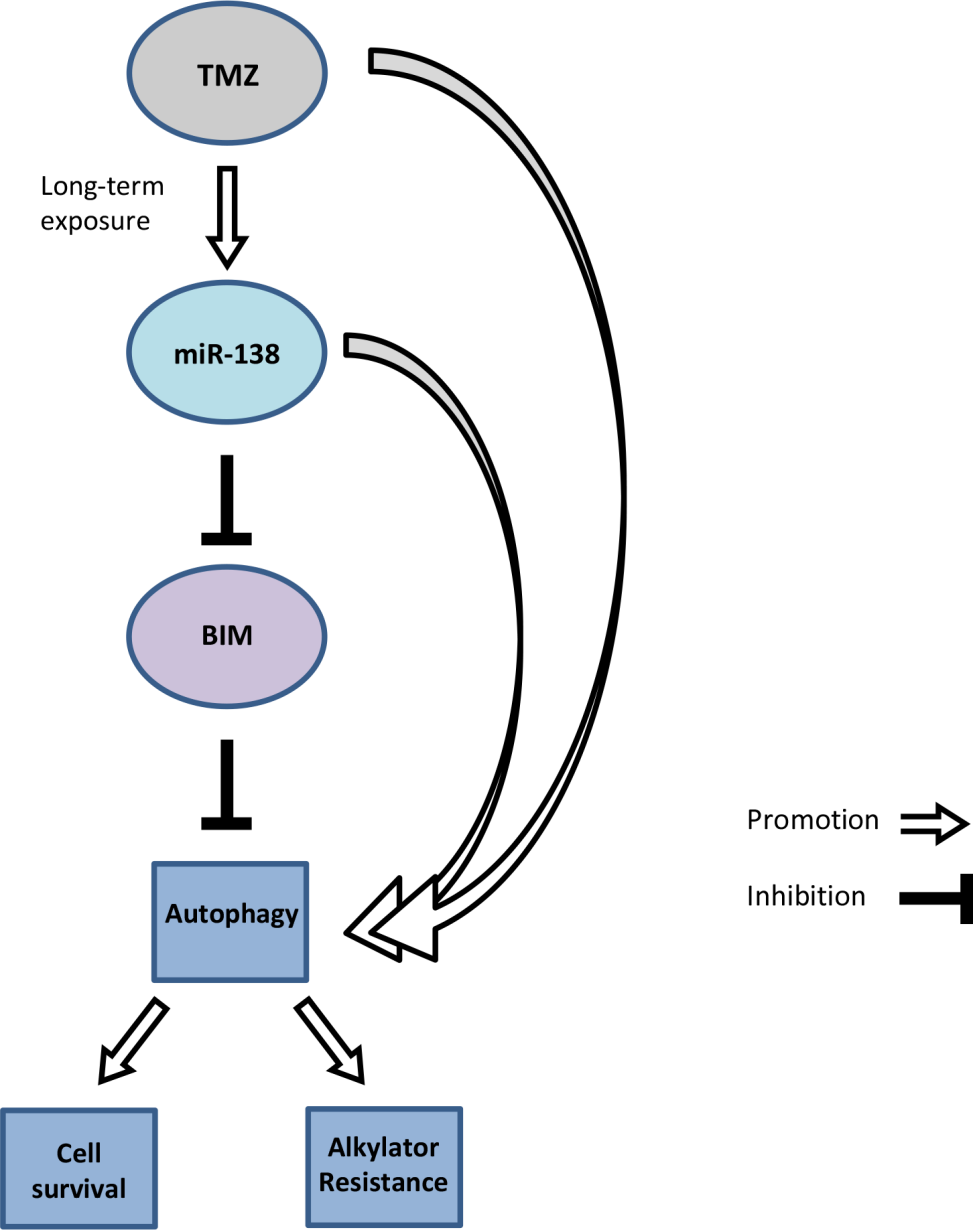
Autophagy mediates TMZ resistance via the miR-138/BIM axis Long-term TMZ treatment induces miR-138 expression, which in turn suppresses BIM translation. Hence, BIM-mediated autophagy inhibition is blocked, and increased autophagy promotes cell survival of the glioma cells.

Consistent with our observation in primary tumors *in vivo* (Figure 1E), transfection of glioma cell lines or glioma stem-like cells with miR-138 mimics increased TMZ resistance (Figure 3A); yet, this effect was only seen in models with low baseline miR-138 expression. Consistently, there was cross-resistance to another alkylating drug, CCNU, but not to irradiation or other chemotherapeutic agents such as vincristine or etoposide, confirming a specific role of this miRNA in the resistance pathway to alkylator-induced DNA damage (Figure 3B; Figure S2B). One challenge that we faced while studying the role of miR-138 was that miR-138 depletion by an inhibitor approach did not show the opposite effect of miR-138 over-expression by adding mimic (Figure S3). We may have encountered this problem due to a technical limitation of the used miRNA silencing approach: miRNA inhibitors are designed as microRNA antisense oligonucleotides, which are supposed to saturate endogenous levels of miRNA by binding them, thus preventing the binding to the target mRNA molecules. However, if the number of antisense oligonucleotides is not in several-fold excess of the miRNA copies per cell, the miRNA saturation is not complete, hence the residual levels that we observe upon miR-138 silencing (Figure S3A) might be sufficient to maintain the original phenotype [38, 39]. We also investigated potential mechanisms that could underlie the induction of TMZ resistance by miR-138. For this purpose, we studied two predicted miR-138 targets for their involvement in TMZ resistance of glioma cells, namely CD166/ALCAM and BCL2L11/BIM. Expression of ALCAM on glioma stem cells has been previously linked to the regulation of glioblastoma invasion [40]. Moreover, RNAi-mediated silencing of ALCAM induced chemotherapy resistance in pancreatic cancer cells [41]. However, while we found decreased ALCAM expression levels in TMZ-resistant cells, our functional analyses did not confirm an effect of ALCAM downregulation on glioma cell sensitivity to TMZ (Figure S4D). In contrast, BIM, a pro-apoptotic BH3-only protein of the Bcl-2 family emerged as an interesting target to study. We succeeded to confirm the *in silico* predictions that BIM is a direct target of miR-138 by luciferase reporter assays (Figure 4C). We further show that transient knockdown of *BIM* increased TMZ resistance of glioma cells (Figure 4E), thus mimicking the miR-138 over-expression phenotype. Therefore, we postulate that the resistance phenotype mediated by miR-138 is partly dependent on BIM downregulation. Two other microRNAs, miR-17 and miR-20a, have been previously shown to target BIM, and their suppression sensitized chemoresistant leukemia cells to DNA damage-induced apoptosis [42]. MiR-148a has also been shown to target BIM in glioma stem cells and thereby control cell proliferation and apoptosis [43]. However, the contribution of miR-mediated BIM regulation to the response of glioma cells to TMZ has not been addressed before. We observed that BIM silencing led to increased TMZ resistance, which was reversed by adding sub-lethal concentrations of the BH3-mimetic ABT-737 (Figure 4F). All experiments assessing TMZ resistance employed clonogenic survival assays, which measure the potential of single cells to form colonies over long periods of time (up to 20 days) upon different stimulations. Since BIM regulated resistance to TMZ, we measured TMZ-induced apoptosis by AnnexinV/PI (AV/PI) assay and detection of cleaved caspase 3. Our flow cytometry AV/PI measurements (Figure 5A) revealed a minor induction of apoptosis 72 h after TMZ treatment (up to 10%) as described before [27, 44]. However, these studies did not investigate caspase 3 cleavage after TMZ exposure of glioma cells, but only after alkylating MNNG treatment, reporting cleavage at 96 h post treatment. Yet, LN-308 cells stimulated with TMZ did not exhibit caspase 3 cleavage at early or later time-points (Figure 5B). Literature reports suggest that TMZ can induce autophagy or senescence rather than apoptosis [27, 45–47]. These studies offer conflicting conclusions whether autophagy is a pro- or anti-survival mechanism. Indeed, autophagy as a mechanism for maintenance of cellular homeostasis can have tumor-suppressive properties in healthy cells. In neoplastic cells, however, autophagic responses rather protect from intracellular and environmental stress, thus promoting tumor progression and resistance to therapy [29]. We indeed saw induction of autophagy after TMZ treatment at day 1 up to day 8 (Figure 5C). This consequence of TMZ exposure was accompanied by reduction of the pro-apoptotic protein BIM and increase of anti-apoptotic Bcl-2 family proteins (Figure 5B). Interestingly, BIM has previously been described to inhibit autophagy by binding and mislocalizing Beclin 1, an autophagy regulator [48]. Additionally, increased autophagy was seen in TMZ-resistant cells, and upon miR-138 over-expression or BIM silencing to a lesser extent (Figure 5C). Finally, blocking autophagy by the inhibitor 3-MA led to increased TMZ sensitivity (Figure 5D). It is tempting to speculate that TMZ treatment, leading to increased miR-138 expression, which in turn reduces BIM levels, leads to autophagy that results in cell survival, thus TMZ resistance. However, probably additional TMZ-induced mechanisms further regulate autophagy (Figure 6).

In conclusion, we demonstrate a role for miR-138 in acquired TMZ resistance in glioma cells. Moreover, we establish a functional link between miR-138-mediated resistance and BIM, and confirm that down-regulation of BIM by miR-138 promotes TMZ resistance. Additionally, we propose that the miR-138/BIM axis mediates resistance to TMZ by autophagy. Hence, its targeting may represent a novel strategy to overcome acquired TMZ resistance in glioblastoma.

## MATERIALS AND METHODS

### Cells and reagents

The human long-term glioma cell lines (LTC) LN-18, LN-229, LN-308, LN-319, LN-428, D247MG, A172, U87MG and T98G were kindly provided by Dr. N. de Tribolet (Lausanne, Switzerland). TMZR sublines (LN-18_R, LN-229_R and LN-308_R) have been characterized [14]. The following glioma-initiating cells (GIC) grown as sphere cultures were used: ZH-161, ZH-305, T-325, T-269 and S-24 [49]. Stock solutions of TMZ (Schering-Plough, Kenilworth, NJ) and lomustine (CCNU) (Dr. Reddy's Laboratories Ltd., Hyderabad, India) at 100 mM were prepared in dimethylsulfoxide (DMSO); aliquots were stored at −20°C. Etoposide (Sigma-Aldrich, St. Louis, MO) stock solution was prepared in DMSO at 50 mM, ABT-737 (ChemieTek, Indianapolis, IN) was prepared as 50 mM stock solution in DMSO. Vincristine was purchased from Tocris Bioscience (Bristol, UK) and prepared as 48 mM stock solution in DMSO. 3-methyl adenine (3-MA) (Sigma solutions) were freshly prepared in water at 100 mM. Irradiation experiments were performed by irradiation of the cells in a Co-radiation source (Gebrüder Sulzer, Thermische Energiesysteme, 60-Co, Winterthur, Switzerland).

### MiRNA and mRNA extraction from glioblastoma tissue samples and glioma cells

Deep-frozen tissue samples from primary and recurrent glioblastomas of 10 patients treated with TMZ/RT→TMZ after surgery were selected from the tumor tissue bank of the Department of Neuropathology, Heinrich Heine University, Düsseldorf, Germany, and investigated as approved by the institutional review board (study number 3825). Tissue samples were snap frozen immediately after resection and stored at −80°C. Each tissue specimen was histologically evaluated for sufficient tumor cell content. miRNA was extracted from frozen tissue samples or cell Qiagen, Hilden, Germany). Total RNA was prepared using the NucleoSpin System (Macherey-Nagel AG, Oensingen SO, Switzerland). RNA quantity was determined with the NanoDrop ND-1000 spectrophotometer (PeqLab, Erlangen, Germany). miRNA and mRNA quality assessment was done with an Agilent 2100 bioanalyzer (Agilent Technologies, Santa Clara, CA). Only RNAs with a RNA integrity number of ≥ 7 were used for molecular analyses.

### Microarray-based miRNA and mRNA expression profiling

For microarray-based miRNA expression analysis 400 ng of high-molecular weight RNA was biotin-labelled using the Affymetrix^®^ FlashTag™ Biotin HSR RNA Labeling Kit (Affymetrix, Santa Clara, CA) and hybridized to GeneChip^®^ miRNA 2.0 arrays (Affymetrix). Transcriptome-wide changes in gene expression were determined by hybridization to Gene Chip^®^ Human Genome U133 Plus 2.0 arrays (Affymetrix) [50]. Sample preparation for mRNA expression analysis was done with 2.5 μg of total RNA using the One Cycle Target Labelling and Controls kit (Affychip hybridizations were performed at the Center for Biological and Medical Research (BMFZ) at Heinrich Heine University Düsseldorf using a GeneChip Hybridization Oven 645 and a GeneChip^®^ Scanner 3000 7G. Three independent batches from each parental and TMZR cell line were subjected to the Affymetrix chip analyses.

### Bioinformatic and statistical analyses of microarray data

All steps of the microarray analysis pipeline were set up within the R framework for statistical computing [51]. The microarray dataset was normalized and log_2_-transformed using the robust multi-array average (RMA) method implemented in the *affy* package [52]. Differential expression of miRNA was assessed by computing the moderated *t*-statistics using the LIMMA package [53]. The resulting *p*-values were corrected for multiple testing by controlling the false discovery rate [54]. A miRNA was considered to be differentially expressed if the corrected *p*-value was below 0.05. For *in silico* predictions of potential miRNA targets, the miRWalk online database on predicted and validated miRNA targets was used [21].

### Transfection reagents, miRNA mimics and inhibitors, and siRNA molecules

The miRIDIAN miR-138 mimics and miR-138 miRIDIAN hairpin inhibitors and respective control molecules were purchased from Dharmacon (Dharmacon GE Healthcare, Lafayette, CO). For *ALCAM* knockdown ON-TARGET plus siRNA-SMART pool (L-004574) was used (Dharmacon). BIM gene silencing was performed using FlexiTube siRNA (siBim1-SI02655359, siBim2 – SI03056466, siBim3 – SI03062080, siBim4 – SI04951961; Qiagen, Hilden, Germany). Transient transfection of glioma cells with mimics, inhibitors or siRNA was performed using Lipofectamine RNAiMAX (Life Technologies, Carlsbad, CA, USA). Lipofectamine 2000 was used for co-transfection of luciferase reporter vector and miRNA mimics or inhibitor molecules (Life Technologies).

### Transient transfection

LTC were seeded in six-well plates at 250,000 to 600,000 cells/well. Cells were transfected with specific miRNA mimics, miRNA inhibitors, siRNA, or respective control molecules 24 h later. For clonogenic assays and mRNA or miRNA expression analyses, cells were harvested 24 h post transfection, while for preparation of protein lysates, medium was changed 24 h post transfection and the cells were harvested 48 h later.

GICs were dissociated using Accutase cell detachment solution (PAA Laboratories; Pasching, Austria). Two million cells were transfected using the Neon Transfection System electroporation device and kit (Invitrogen, Life Technologies) with 100 nM mimic, inhibitor, siRNA, or control. After transfection, cells were seeded in 6-well plates in neurobasal conditions for recovery. One day later cells were processed for clonogenic survival assay, mRNA or microRNA isolation, or 72 h later for protein lysate preparation.

### Quantitative reverse transcriptase PCR

Complementary DNA transcription from miR-138 and RNU48 and subsequent real-time PCR-FAM detection was performed using specific TaqMan™ MicroRNA Assays (hsa-miR-138 assay ID: #002284; RNU48 assay ID: #001006) (Applied Biosystems, Life Technologies). For mRNA expression analyses, total RNA was transcribed into cDNA using the iScript cDNA Synthesis Kit (Bio-Rad Laboratories; Hercules, CA). cDNA amplification was monitored using SYBRGreen chemistry on the 7300 Real time PCR System (Applied Biosystems). Conditions for PCR reactions were: 40 cycles, 95°C/15 sec, 60°C/1 min, using the following specific primers: *GAPDH* fwd: 5′-CTCTCTGCTCCTCCTGTTCGAC-3′ (NM_002046, 114-135 nt), *GAPDH* rv: 5′-TGAGCGATGTGGCTCGGCT-3′ (NM_002046, 182-164 nt), *ALCAM* fwd: 5′-TTCTTAGCACCTGGCGTTTC-3′ (NM_001627, 293-312 nt), *ALCAM* rv: 5′-CGGTTCTTTTCGCTGGTATC-3′ (NM_001627, 388-369 nt), *BIM* fwd: 5′-TTGATTCTTGCAGCCACCCT-3′ (NM_138621, 120-139 nt), *BIM* rv: 5′-GAACGCAGCGAACCGAATAC-3′ (NM_138621, 193-174 nt). Data analysis was done using the ΔC_T_ method for relative quantification, *GAPDH* and *RNU48* were used as control genes for mRNA or miRNA expression [55].

### miRNA expression analyses in glioma tissue specimens

Reverse transcription of mature miR-138 in primary and recurrent glioblastomas was carried out with 100 ng of total RNA as template using stem-loop primers specific for miR-138 and Superscript^®^ II reverse transcriptase (Life Technologies). Real-time PCR on the StepOnePlus™ system (Life Technologies) was done with TaqMan^®^ miRNA assays (Applied Biosystems, Life Technologies) for miR-138 (Assay ID: #002284) and *U6 snRNA* (Assay ID: #001973) as reference. Expression changes relative to a calibrator sample (Universal Human Reference RNA, Stratagene, La Jolla, CA) were calculated with the 2^−ΔΔ Ct^ method [56].

### Immunoblotting

Immunoblot analysis was performed as described previously [57]. The following primary antibodies were used: anti-β-actin (csc-1616) (Santa Cruz Biotechnology, Dallas, TX), anti-SOX4 antibody (ab41891) (Abcam, Cambridge, UK), anti-ALCAM antibody (MAB656) (R & D Systems, Minneapolis, MN) anti-Cleaved Caspase-3 (Asp175) (#9661), anti-Bcl-xL (#2764) and anti-LC3 A/B (#4108) (Cell Signaling Technology, Danvers, MA), and anti-BIM antibody (B7929) (Sigma-Aldrich).

### Proliferation, viability, clonogenicity and flow cytometry

BrdU cell proliferation assays were performed using a Cell Signaling Technology kit (Danvers, MA). Briefly, 24 h after miRNA mimic or control transfection, the cells were seeded at 5,000 cells/well in 96-well plates. BrdU reagent was added 24 h later in serum-free DMEM medium, and cells were grown for another 24 h. BrdU detection was performed according to the user's manual. Trypan blue dye exclusion assay was also used to monitor cell viability. Transfected cells were seeded at a density of 5,000 cells/well in 12-well plates. Medium was changed to serum-free DMEM 24 h later for specified experiments; after 24 h floating and attached cells were harvested, and living and dead cells were counted in a Neubauer chamber using 0.4% trypan blue. Clonogenic assays for adherent LTC and GIC cultures were performed as described previously [14, 50]. For cell cycle and cell death analysis, transfected LN-308 cells were treated as indicated, harvested at the indicated time points after transfection (cell cycle analysis) or post-treatment (cell death analysis) and analyzed [14].

### 3′UTR luciferase gene reporter assay

A 1281-bp fragment of the *BIM* 3′UTR sequence containing two predicted binding sites for miR-138 was subcloned into the psiCHECK™-2 dual luciferase vector (Promega, *Madison, WI*). For this purpose cDNA from LN-308 cells was used and the following primers for amplification of the insert: fwd: 5′-CTCGAGATTATGCAGCCAGCGGTTCT-3′, rev: 5′-GCGGCCGCAGCAGGCACAGAGAAAGAGC-3′. Restriction enzymes used for cloning the insert 3′ to the Renilla luciferase were XhoI and NotI (New England Biolabs, Ipswich, MA). For the control vectors the same fragment of *BIM* 3′UTR sequence was synthesized *de novo*, bearing mutations in the two miR-138 recognition sites: psiCheck2_mut1 (mutations in site 161-185 nt); psiCheck2_mut2 (mutations in site 1223-1249 nt); psiCheck2_mut3 (mutations in both recognition sites) (Eurofins Genomics, Ebersberg, Germany). Dual luciferase reporter assays were performed seeding 20,000 LN-308 cells per well in a 96-well plate. Following attachment overnight, cells were co-transfected with 100 ng of the respective reporter construct and 50 nM of miR-138 mimic or control molecules. After 48 h, cells were lysed, luciferase activity was measured, and Renilla values normalized to *Firefly* luciferase.

### The cancer genome atlas (TCGA) database

Data was obtained from the glioblastoma data set of the Cancer Genome Atlas network (http://cancergenome.nih.gov/) [22]. The affymetrix probesets used in the TCGA database were 201952_at for ALCAM and 208536_s_at for BCL2L11. Survival analysis within the glioblastoma data set of the TCGA database was performed using the Kaplan-Meier analysis module of the R2 microarray analysis and visualization platform (http://r2.amc.nl).

### Statistical analyses

Data are expressed as means and standard deviations. The experiments shown were independently repeated a minimum of three times with similar results. If not otherwise indicated, results of representative experiments are shown. Significance was assessed using two-sided unpaired Student's *t*-test, paired Student's *t*-test for comparison of miR-138 expression in primary and recurrent glioblastomas, one-sample Student's *t*-test for analysis of luciferase reporter results or two-way ANOVA with post hoc Bonferroni test, and correlation was calculated by assessing the Spearman's coefficient (r) (GraphPad Prism 5, La Jolla, CA) (*p < 0.05, **p < 0.01, ***p < 0.001).

## SUPPLEMENTARY MATERIALS FIGURES


