# Supplementary material for: MicroRNA-138 promotes acquired alkylator resistance in glioblastoma by targeting the Bcl-2-interacting mediator BIM
Source: Oncotarget. 2016 Feb 12;7(11):12937–50. doi: 10.18632/oncotarget.7346 (PMC4914333; doi:10.18632/oncotarget.7346)
Supplement: Supplementary file 1 [file oncotarget-07-12937-s001.pdf]

MicroRNA-138 promotes acquired alkylator resistance in glioblastoma by targeting the Bcl-2-interacting mediator BIM

Supplementary Materials

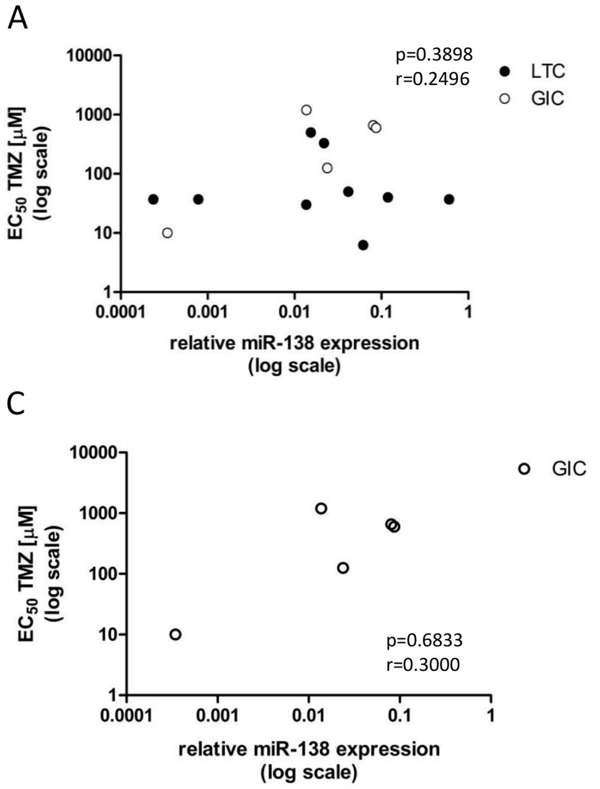

Supplementary Figure S1: Correlation between miR-138 expression and EC_{50} values for TMZ. (**A**) Correlation between EC_{50} values for TMZ and baseline miR-138 expression for 9 LTC and 5 GIC lines, (**B**) for 9 LTC alone assessed by the Spearman's coefficient, and (**C**) for 5 GIC lines (correlation was calculated by assessing the Spearman's coefficient).

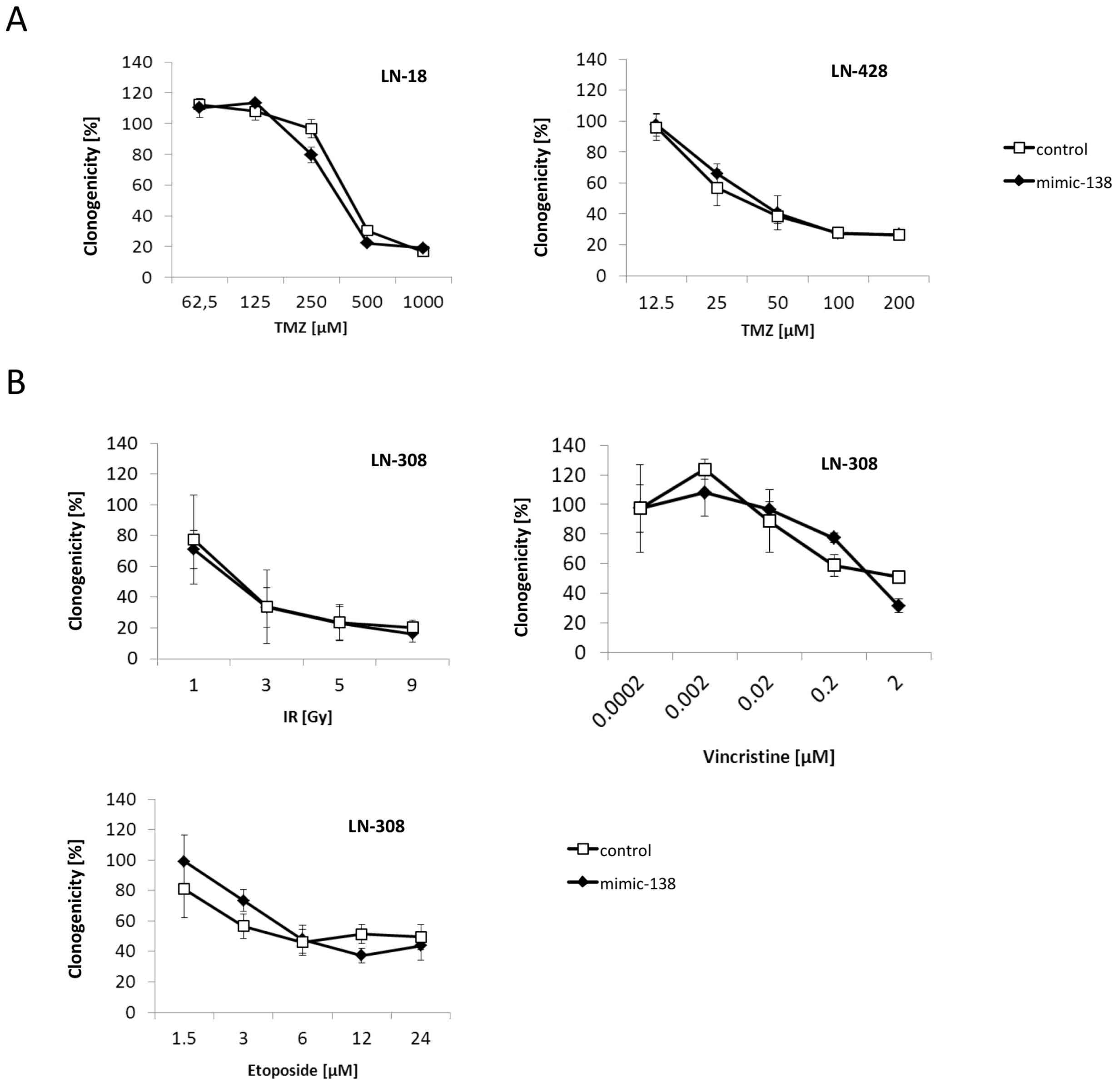

Supplementary Figure S2: Specifity for miR-138-mediated protection from alkylating agents in cell lines with low endogenous miR-138 expression. (**A**) Clonogenic cell survival assay after TMZ treatment of LN-18 and LN-428 cells transfected with miR-138 mimic or control (50 nM). (**B**) Clonogenic cell survival assay after irradiation, vincristine or etoposide treatment of LN-308 cells transfected with miR-138 mimic or control (50 nM). Cells were exposed 24 h after transfection to either chemotherapeutic agent for 24 h, or irradiated at the indicated dose.

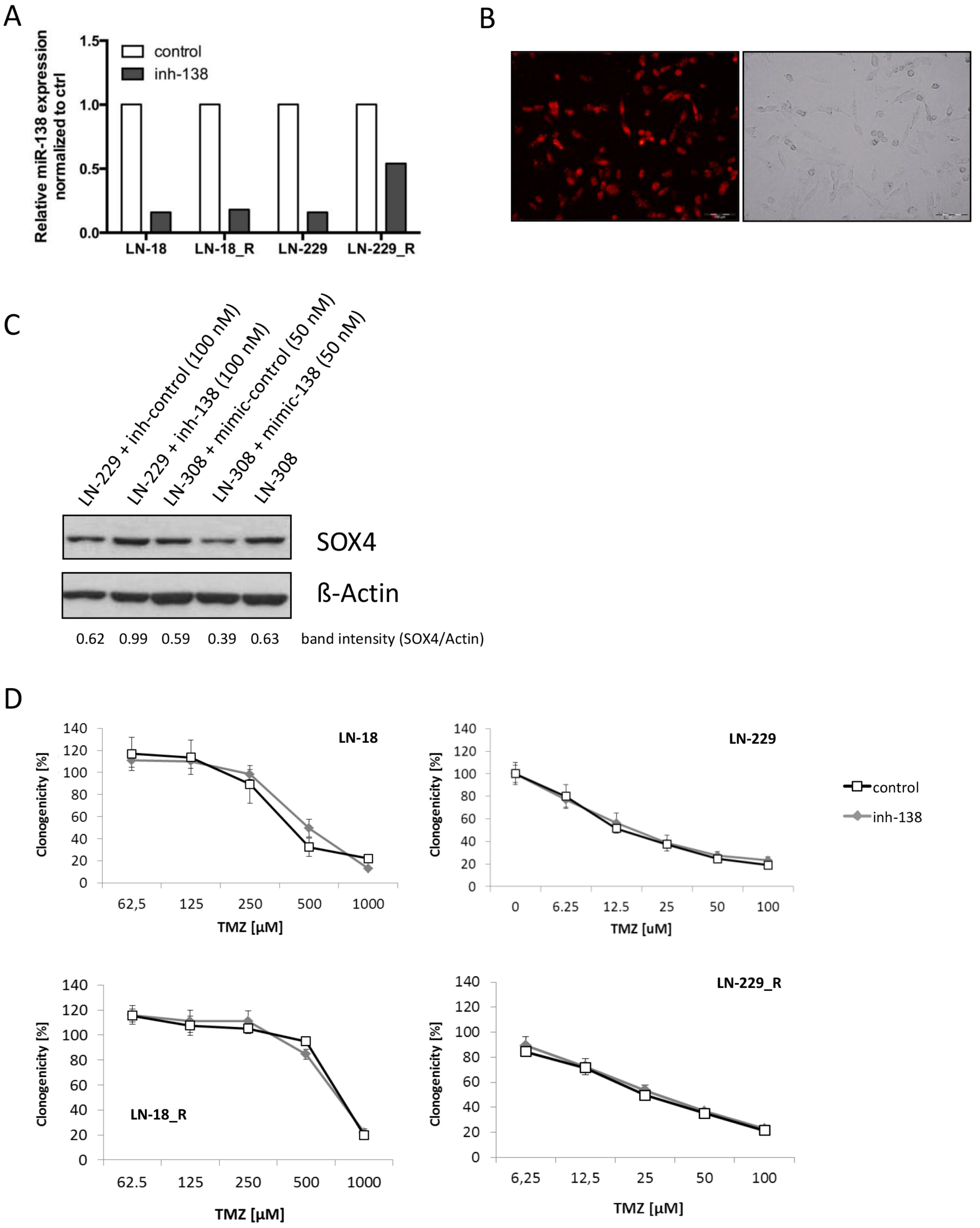

Supplementary Figure S3: miR-138 inhibitor alone is not sufficient to sensitize glioma cells to TMZ. (**A**) miR-138 inhibitor transfection efficacy expressed as fold change ratio of relative expression of miR-138 in inhibitor vs. control transfected cells in LN-18, LN-229, LN-18_R and LN-229_R cells. Relative expression of miR-138 was determined by real-time qPCR. (**B**) miR-138 inhibitor transfection (100 nM) efficiency confirmation by light fluorescence microscopy, inhibitor control molecule labeled with Dy547 visible as red staining. (**C**) Immunoblot of SOX4 levels in glioma LTC lines transfected with miR-138 mimics, inhibitors or respective control molecules. Quantification of protein bands was performed by densitometric analysis, and the values stated under each band represent the normalized value to the corresponding loading control signal. (**D**) Clonogenic cell survival assays of TMZ-treated LN-18, LN-18_R, LN-229 or LN-229_R cells transfected with miR-138 inhibitors or controls.

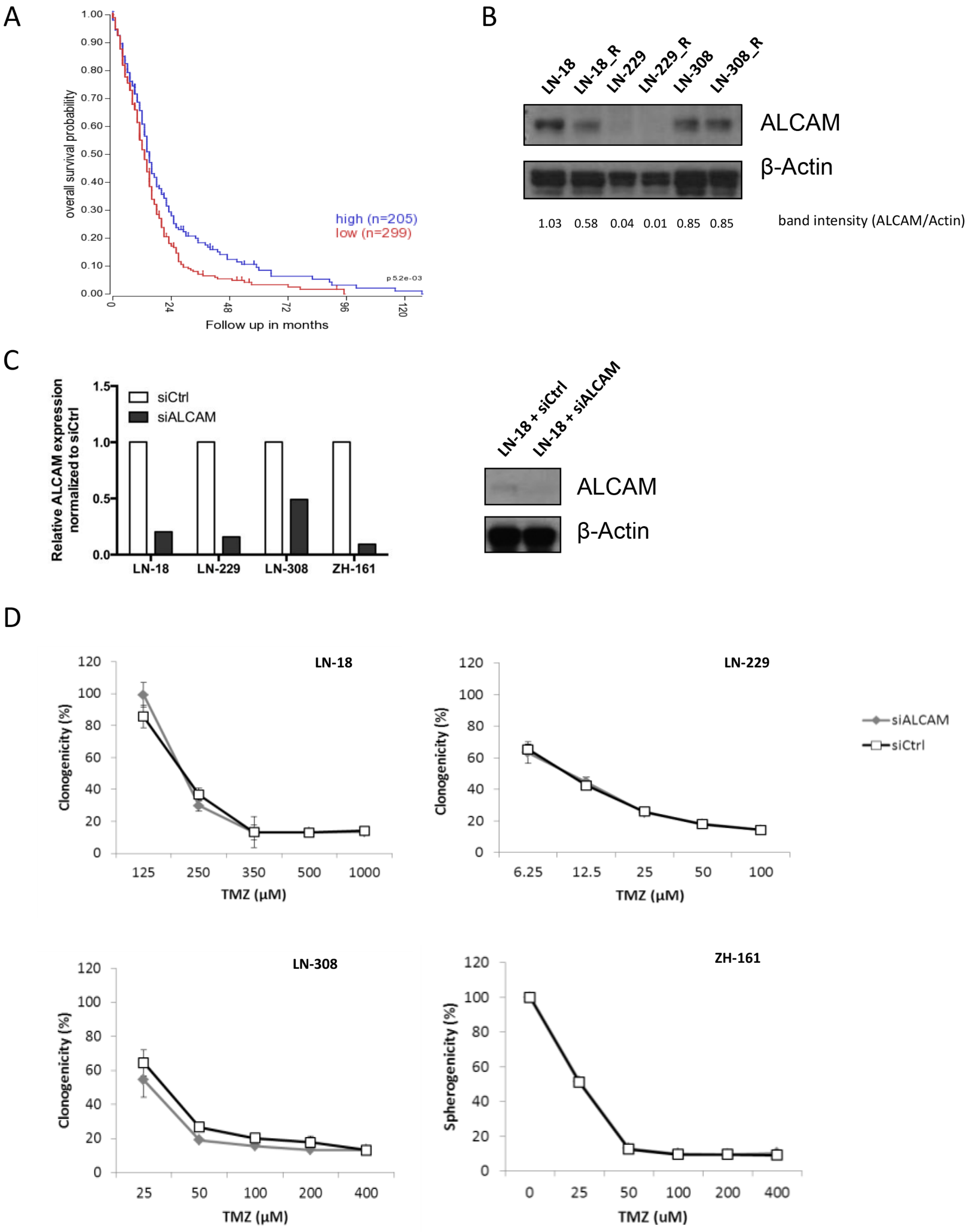

Supplementary Figure S4: ALCAM alone does not mediate TMZ sensitivity in LTC or GIC. (**A**) Association of overall survival probability of glioblastoma patients with low or high expression of ALCAM obtained from the TCGA data set. (**B**) Immunoblot of ALCAM protein levels in parental LN-18, LN-229 or LN-308 cells and R cells. (**C**) Knockdown confirmation of ALCAM by siRNA shown by real-time qPCR (left panel) for LN-18, LN-229, LN-308 or ZH-161 cells, and by immunoblot for LN-18 cells (right panel). (**D**) LN-18, LN-229 or LN-308 LTC, or ZH-161 GIC were transfected with siALCAM or siCtrl and TMZ sensitivity was assessed by clonogenic survival assays in LTC and spherogenicity assay in GIC.

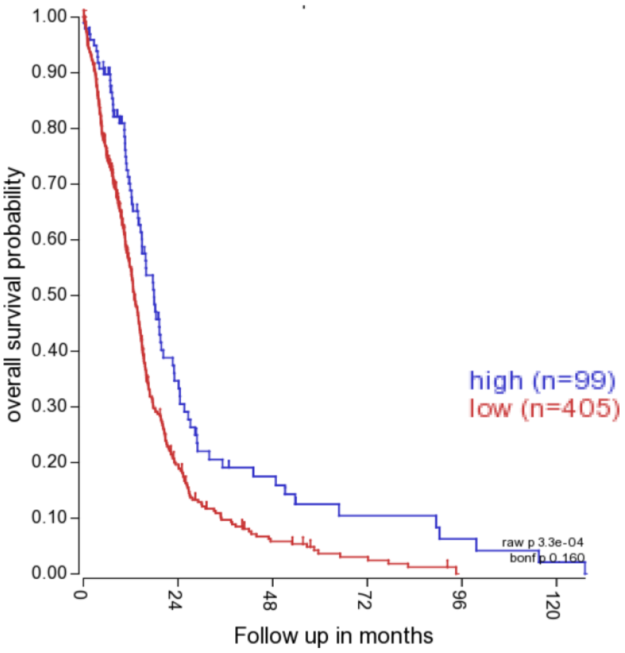

Supplementary Figure S5: TCGA analysis of glioblastoma patients with low or high BIM expression. Association of overall survival probability of glioblastoma patients with low or high expression of BCL2L11/BIM obtained from the TCGA data set.

A

3’ GCC-GGA-CUA-AGU-GUU-GUG-GUC-GA 5’ **hsa-miR-138**

161: 5’ CTT-TCT-GTT-CAT-CAC-CAC-ACA-GCA 3’ **BCL2L11**

161: 5’ CTT-TCT-Gta-gAT-CAC-Ctg-Agt-GgA 3’ **BCL2L11** **mut**

1223: 5’ AGG-CCT-CAT-CCC-ACT-TCC-ACC-AGC-A 3’ **BCL2L11**

1223: 5’ AGG-CCT-CAT-CCg-tCT-TCg-Agg-caC-A 3’ **BCL2L11 mut**

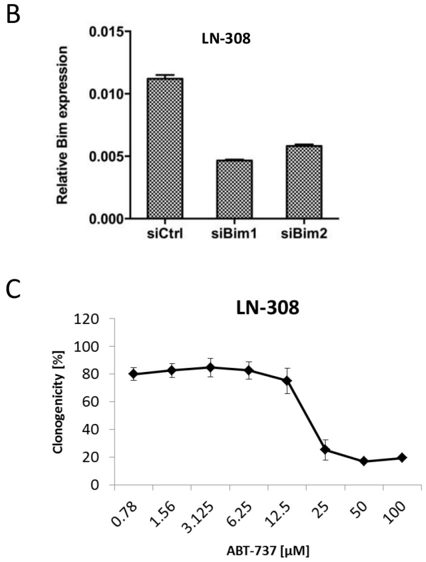

Supplementary Figure S6: Predicted miR-138 binding sites in BIM 3’UTR region, knockdown efficiency of *BIM* and ABT-737 toxicity assessment. (**A**) *In silico* predicted interactions between miR-138 and the 3’UTR region of BIM, and the mutations in both miR-138 binding sites present in the mutated luciferase reporter assay control vectors; mutated nucleotides shown in small letters (**B**) siRNA-mediated silencing of *BIM* using two different constructs in LN-308 cells shown at the RNA level, assessed by real-time qPCR. (**C**) ABT-737 EC_{50} values were assessed in LN-308 cells by clonogenic survival assay.
